# Global Spore Sampling Project: A global, standardized dataset of airborne fungal DNA

**DOI:** 10.1038/s41597-024-03410-0

**Published:** 2024-05-30

**Authors:** Otso Ovaskainen, Nerea Abrego, Brendan Furneaux, Bess Hardwick, Panu Somervuo, Isabella Palorinne, Nigel R. Andrew, Ulyana V. Babiy, Tan Bao, Gisela Bazzano, Svetlana N. Bondarchuk, Timothy C. Bonebrake, Georgina L. Brennan, Syndonia Bret-Harte, Claus Bässler, Luciano Cagnolo, Erin K. Cameron, Elodie Chapurlat, Simon Creer, Luigi P. D’Acqui, Natasha de Vere, Marie-Laure Desprez-Loustau, Michel A. K. Dongmo, Ida B. Dyrholm Jacobsen, Brian L. Fisher, Miguel Flores de Jesus, Gregory S. Gilbert, Gareth W. Griffith, Anna A. Gritsuk, Andrin Gross, Håkan Grudd, Panu Halme, Rachid Hanna, Jannik Hansen, Lars Holst Hansen, Apollon D. M. T. Hegbe, Sarah Hill, Ian D. Hogg, Jenni Hultman, Kevin D. Hyde, Nicole A. Hynson, Natalia Ivanova, Petteri Karisto, Deirdre Kerdraon, Anastasia Knorre, Irmgard Krisai-Greilhuber, Juri Kurhinen, Masha Kuzmina, Nicolas Lecomte, Erin Lecomte, Viviana Loaiza, Erik Lundin, Alexander Meire, Armin Mešić, Otto Miettinen, Norman Monkhause, Peter Mortimer, Jörg Müller, R. Henrik Nilsson, Puani Yannick C. Nonti, Jenni Nordén, Björn Nordén, Claudia Paz, Petri Pellikka, Danilo Pereira, Geoff Petch, Juha-Matti Pitkänen, Flavius Popa, Caitlin Potter, Jenna Purhonen, Sanna Pätsi, Abdullah Rafiq, Dimby Raharinjanahary, Niklas Rakos, Achala R. Rathnayaka, Katrine Raundrup, Yury A. Rebriev, Jouko Rikkinen, Hanna M. K. Rogers, Andrey Rogovsky, Yuri Rozhkov, Kadri Runnel, Annika Saarto, Anton Savchenko, Markus Schlegel, Niels Martin Schmidt, Sebastian Seibold, Carsten Skjøth, Elisa Stengel, Svetlana V. Sutyrina, Ilkka Syvänperä, Leho Tedersoo, Jebidiah Timm, Laura Tipton, Hirokazu Toju, Maria Uscka-Perzanowska, Michelle van der Bank, F. Herman van der Bank, Bryan Vandenbrink, Stefano Ventura, Solvi R. Vignisson, Xiaoyang Wang, Wolfgang W. Weisser, Subodini N. Wijesinghe, S. Joseph Wright, Chunyan Yang, Nourou S. Yorou, Amanda Young, Douglas W. Yu, Evgeny V. Zakharov, Paul D. N. Hebert, Tomas Roslin

**Affiliations:** 1 Department of Biological and Environmental Science, University of Jyväskylä, P.O. Box 35, FI-40014 Jyväskylä, Finland; 2 Organismal and Evolutionary Biology Research Programme, Faculty of Biological and Environmental Sciences, University of Helsinki, P. O. Box 65, 00014 Helsinki, Finland; 3 Department of Biology, Centre for Biodiversity Dynamics, Norwegian University of Science and Technology, Trondheim, N-7491 Norway; 4 Department of Agricultural Sciences, University of Helsinki, P.O. Box 27, FI-00014 Helsinki, Finland; 5 Natural History Museum, Zoology, University of New England, Armidale, NSW 2351 Australia; 6 Faculty of Science and Engineering, Southern Cross University, Northern Rivers, NSW 2480 Australia; 7 Wrangel Island State Nature Reserve, Pevek, Russia; 8 Department of Biological Sciences, MacEwan University, 10, 700 – 104 Avenue, Edmonton, AB T5J 2P2 Canada; 9 Universidad Nacional de Còrdoba, Facultad de Ciencias Exactas Físicas y Naturales, Centro de Zoología Aplicada, Córdoba, Argentina; 10 Sikhote-Alin State Nature Biosphere Reserve named after K. G. Abramov, 44 Partizanskaya Str., Terney, Primorsky krai 692150 Russia; 11 School of Biological Sciences, The University of Hong Kong, Hong Kong SAR, China; 12 CSIC, Institute of Marine Sciences, Passeig Marítim de la Barceloneta, 37-49ES08003 Barcelona, Spain; 13 Institute of Arctic Biology, University of Alaska, Fairbanks, AK USA; 14 Goethe-University Frankfurt, Faculty of Biological Sciences, Institute for Ecology, Evolution and Diversity, Conservation Biology, D- 60438 Frankfurt am Main, Germany; 15 Bavarian Forest National Park, Freyunger Str. 2, D-94481 Grafenau, Germany; 16 Ecology of Fungi, Bayreuth Center of Ecology and Environmental Research (BayCEER), University of Bayreuth, Universitätsstraße 30, 95440 Bayreuth, Germany; 17 Consejo de Investigaciones Científicas y Técnicas (CONICET), Instituto Multidisciplinario de Biología Vegetal, Córdoba, Argentina; 18 Department of Environmental Science, Saint Mary’s University, 923 Robie St., Halifax, NS B3H 3C3 Canada; 19 Department of Ecology, Swedish University of Agricultural Sciences (SLU), Uppsala, Sweden; 20 Molecular Ecology and Evolution at Bangor (MEEB), School of Environmental and Natural Sciences, Bangor University, Environment Centre Wales, Deiniol Road, Bangor, Gwynedd, Wales LL57 2UW UK; 21 Research Institute on Terrestrial Ecosystems - IRET, National Research Council - CNR, Via Madonna del Piano n° 10, 50019 Sesto Fiorentino Firenze, Italy; 22 National Biodiversity Future Center, Palermo, Italy; 23 Natural History Museum of Denmark, University of Copenhagen, Gothersgade 130, 1123 København K, Denmark; 24 INRAE, BIOGECO, F-33610 Cestas, France; 25 University of Bordeaux, BIOGECO, F-33615 Bordeaux, France; 26 International Institute of Tropical Agriculture (IITA), P.O. Box 2008 (Messa), Yaoundé, Cameroon; 27 Greenland Institute of Natural Resources, Kivioq 2, P.O. Box 570, 3900 Nuuk, Greenland; 28 Entomology, 55 Music Concourse Drive, California Academy of Sciences, San Francisco, CA 94118 USA; 29 Madagascar Biodiversity Center, Parc Botanique et Zoologique de Tsimbazaza, Antananarivo, 101 Madagascar; 30 Legado das Águas, Reserva Votorantin, TPR 188 Km 22, Tapiraí, SP 18180-000 Brazil; 31 Environmental Studies Department, University of California, Santa Cruz, 1156 High St., Santa Cruz, CA 95065 USA; 32 Department of Life Sciences, Aberystwyth University, Aberystwyth, Ceredigion WALES SY23 3DD UK; 33 Research Unit Biodiversity and Conservation Biology, SwissFungi, Swiss Federal Research Institute WSL, Zürcherstrasse 111, CH-8903 Birmensdorf, Switzerland; 34 Swedish Polar Research Secretariat, Abisko Scientific Research Station, Vetenskapens väg 38, SE-981 07 Abisko, Sweden; 35 Center for Tropical Research, Congo Basin Institute, University of California, Los Angeles (UCLA), Los Angeles, CA 90095 USA; 36 Department of Ecoscience, Aarhus University, Dk-4000 Roskilde, Denmark; 37 Research Unit in Tropical Mycology and Plant-Soil Fungi Interactions, Faculty of Agronomy, University of Parakou, BP 123, Parakou, Republic of Benin; 38 Canadian High Arctic Research Station, Polar Knowledge Canada, PO Box 2150, 1 Uvajuq Road, Cambridge Bay, Nunavut X0B 0C0 Canada; 39 Department of Integrative Biology, College of Biological Science, University of Guelph, 50 Stone Road East, Guelph, Ontario N1G 2W1 Canada; 40 School of Science, University of Waikato, Private Bag 3105, Hamilton, 3240 New Zealand; 41 Department of Microbiology, University of Helsinki, Viikinkaari 9, FI-00014 Helsinki, Finland; 42 Natural Resources Institute Finland, Latokartanonkaari 9, 00790 Helsinki, Finland; 43 Center of Excellence in Fungal Research, Mae Fah Luang University, Chiang Rai, 57100 Thailand; 44 Pacific Biosciences Research Center, University of Hawaii at Manoa, Honolulu, HI USA; 45 Centre for Biodiversity Genomics, University of Guelph, Guelph, ON N1G 2W1 Canada; 46 Nature Metrics North America Ltd., 590 Hanlon Creek Boulevard, Unit 11, Guelph, ON N1C 0A1 Canada; 47 Plant Pathology Group, Institute of Integrative Biology, ETH Zurich, Zurich, Switzerland; 48 Plant Health, Natural Resources Institute Finland (Luke), Jokioinen, Finland; 49 Science Department, National Park Krasnoyarsk Stolby, 26a Kariernaya str., 660006 Krasnoyarsk, Russia; 50 Institute of Ecology and Geography, Siberian Federal University, 79 Svobodny pr., 660041 Krasnoyarsk, Russia; 51 Department of Botany and Biodiversity Research, University of Vienna, Rennweg 14, 1030 Wien, Austria; 52 Centre d’études nordiques and Canada Research Chair in Polar and Boreal Ecology, Department of Biology, Pavillon Rémi-Rossignol, 18, Antonine-Maillet, Université de Moncton, Moncton, NB E1A 3E9 Canada; 53 Department of Evolutionary Biology and Environmental Sciences, University of Zürich, Zürich, Switzerland; 54 Laboratory for Biological Diversity, Rudjer Boskovic Institute, Bijenicka cesta 54, HR-10000 Zagreb, Croatia; 55 Finnish Museum of Natural History, University of Helsinki, P.O. Box 7, 00014 Helsinki, Finland; 56 Centre for Mountain Futures, Kunming Institute of Botany, Chinese Academy of Sciences, Kunming, China; 57 Field Station Fabrikschleichach, Department of Animal Ecology and Tropical Biology (Zoology III), Julius Maximilians University Würzburg, Rauhenebrach, Germany; 58 Bavarian Forest National Park, Grafenau, Germany; 59 Department of Biological and Environmental Sciences, Gothenburg Global Biodiversity Centre, University of Gothenburg, Box 461, 405 30 Göteborg, Sweden; 60 Norwegian Institute for Nature Research (NINA), Sognsveien 68, N-0855 Oslo, Norway; 61 Department of Biodiversity, Institute of Biosciences, São Paulo State University, Av 24A 1515, Rio Claro, SP 13506-900 Brazil; 62 Department of Entomology and Acarology, Laboratory of Pathology and Microbial Control, University of São Paulo, CEP 13418-900 Piracicaba, SP Brazil; 63 Department of Geosciences and Geography, Faculty of Science, University of Helsinki, P.O. Box 64, 00014 Helsinki, Finland; 64 State Key Laboratory for Information Engineering in Surveying, Mapping and Remote Sensing, Wuhan University, Wuhan, 430079 China; 65 Wangari Maathai Institute for Environmental and Peace Studies, University of Nairobi, P.O. Box 29053, 00625 Kangemi, Kenya; 66 Max Planck Institute for Evolutionary Biology, August-Thienemann-Str. 2, 24306 Plön, Germany; 67 School of Science and the Environment, University of Worcester, Henwick Grove, Worcester WR2 6AJ UK; 68 Department of Ecosystem Monitoring, Research & Conservation, Black Forest National Park, Kniebisstraße 67, 77740 Bad Peterstal-Griesbach, Germany; 69 School of Resource Wisdom, University of Jyväskylä, P.O. Box 35, FIN-40014 Jyväskylä, Finland; 70 The Biodiversity Unit of the University of Turku, Henrikinkatu 2, 20500 Turku, Finland; 71 School of Science, Mae Fah Luang University, Chiang Rai, 57100 Thailand; 72 Southern Scientific Center of the Russian Academy of Sciences, 41 Chekhov ave., Rostov-on-Don, 344006 Russia; 73 State Nature Reserve Olekminsky, Olekminsk, Russian Federation Russia; 74 Mycology and Microbiology Center, University of Tartu, Tartu, Estonia; 75 Institute of Ecology and Earth Sciences, University of Tartu, Liivi 2, 50409 Tartu, Estonia; 76 Arctic Research Center, Aarhus University, Dk-4000 Roskilde, Denmark; 77 TUD Dresden University of Technology, Forest Zoology, Pienner Str. 7, 01737 Tharandt, Germany; 78 Technical University of Munich, Terrestrial Ecology Research Group, Department of Life Science Systems, School of Life Sciences, Hans-Carl-von-Carlowitz-Platz 2, 85354 Freising, Germany; 79 Department of Environmental Science, Aarhus University, Frederiksborgvej 399, DK-4000 Roskilde, Denmark; 80 The Biodiversity Unit of the University of Turku, Kevontie 470, 99980 Utsjoki, Finland; 81 College of Science, King Saud University, Riyadh, Saudi Arabia; 82 School of Natural Science and Mathematics, Chaminade University of Honolulu, Honolulu, HI USA; 83 Laboratory of Ecosystems and Coevolution, Graduate School of Biostudies, Kyoto University, Kyoto, 606-8501 Japan; 84 Center for Living Systems Information Science (CeLiSIS), Graduate School of Biostudies, Kyoto University, Kyoto, 606-8501 Japan; 85 African Centre for DNA Barcoding (ACDB), University of Johannesburg, PO BOX 524, Auckland Park, 2006 South Africa; 86 Sudurnes Science and Learning Center, Garðvegi 1, 245 Sandgerði, Iceland; 87 State Key Laboratory of Genetic Resources and Evolution, Kunming Institute of Zoology, Chinese Academy of Sciences, Kunming, China; 88 Smithsonian Tropical Research Institute, Apartado, 0843–03092 Balboa, Panama; 89 School of Biological Sciences, University of East Anglia, Norwich, Norfolk, NR4 7TJ UK; 90 Yunnan Key Laboratory of Biodiversity and Ecological Security of Gaoligong Mountain, Kunming Institute of Zoology, Chinese Academy of Sciences, Kunming, China

**Keywords:** Biodiversity, Community ecology

## Abstract

Novel methods for sampling and characterizing biodiversity hold great promise for re-evaluating patterns of life across the planet. The sampling of airborne spores with a cyclone sampler, and the sequencing of their DNA, have been suggested as an efficient and well-calibrated tool for surveying fungal diversity across various environments. Here we present data originating from the Global Spore Sampling Project, comprising 2,768 samples collected during two years at 47 outdoor locations across the world. Each sample represents fungal DNA extracted from 24 m^3^ of air. We applied a conservative bioinformatics pipeline that filtered out sequences that did not show strong evidence of representing a fungal species. The pipeline yielded 27,954 species-level operational taxonomic units (OTUs). Each OTU is accompanied by a probabilistic taxonomic classification, validated through comparison with expert evaluations. To examine the potential of the data for ecological analyses, we partitioned the variation in species distributions into spatial and seasonal components, showing a strong effect of the annual mean temperature on community composition.

## Background & Summary

Fungi are one of the most diverse and ecologically important yet unexplored kingdoms of life^1^. From a practical perspective, fungi are infamously hard to sample^2^ and characterize^3^. Recent advancements in DNA-based survey methods have revolutionized studies on fungal diversity, especially its large-scale patterns^4–8^. Given that fungi occur in nearly every possible environment and substrate, current sampling campaigns and estimates of fungal diversity tend to rely explicitly on substrate-specific sampling^9^. Sampling of soil has been popular given the relative ease with which the mycobiome of any handful of soil can be characterized through metabarcoding^10^. Yet, whether biogeographic patterns from those substrates broadly reflect patterns in fungal taxa^9^ or biodiversity in general^11^ is unclear. Additionally, there are significant biases in the geographic areas represented in global studies^12,13^, although there have been recent efforts to expand the coverage of understudied regions^10^.

A recent methodological breakthrough for surveying fungi uses a cyclone sampler to capture fungal spores from the air, followed by DNA sequencing and sequence-based species identification^14^. Air sampling has revealed high diversity and stronger ecological signals in community composition of fungi than soil sampling^15^. Air sampling captures any fragments of fungi floating in the air, including the wind-dispersed spores of fungi and fragments of hyphae as well as fungal structures attached to other organisms. Consequently, air sampling detects fungal dispersal at high temporal resolution. In addition to fungal surveys, the sampling of airborne DNA has proved effective in acquiring comprehensive inventories of regional diversity of many other taxa^16^.

**Fig. 1:**
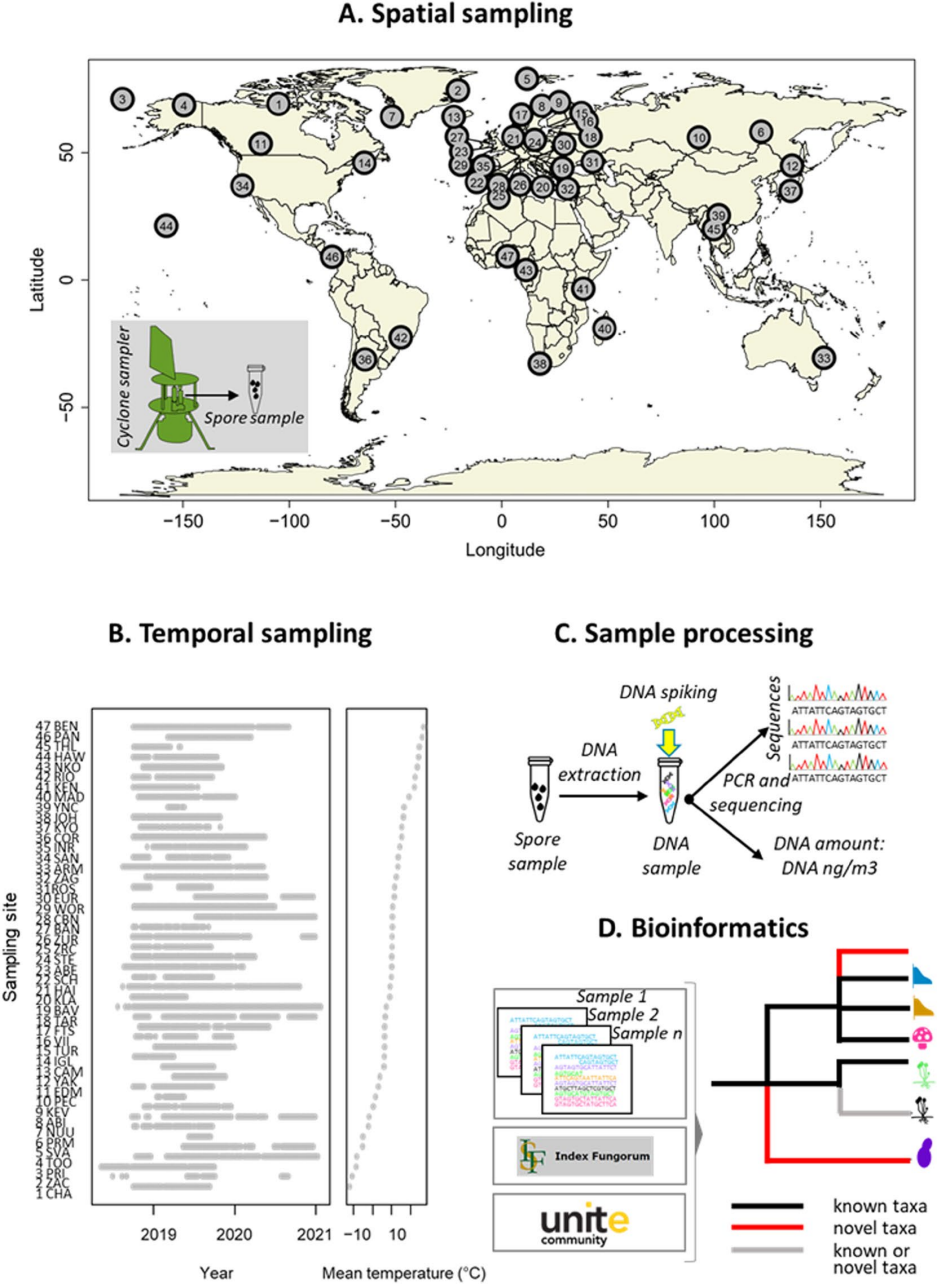
Study design and data generation pipeline of the Global Spore Sampling Project (GSSP). (**A**) The sampling design includes 47 sites with a global distribution, with the greatest coverage in Europe (22 sites) and the poorest coverage in the Southern hemisphere (6 sites). The airborne fungal samples were collected by a cyclone sampler, with each sample consisting of fungal spores filtered from 24 m3 of air during the 24-hr sampling period. (**B**) The study design included weekly samples for a sampling period over one to two years, with some variation among the sites caused mainly by logistical constraints. The sites are ordered according to their mean annual temperature. (**C**) We employed a metabarcoding approach to sequence the fungal ITS2 marker and quantified the amount of fungal DNA (in units of ng of DNA per m3 of air) using a spiking approach17. (**D**) We employed a bioinformatics pipeline that utilized denoising to obtain amplicon sequence variants (ASVs). We then combined probabilistic taxonomic placement with a constrained clustering approach to form species-level OTUs, and to place these OTUs in a taxonomic tree to the most resolved taxonomic level possible given the limitations of sequence reference databases. This tree consists of three types of branches: taxa that could be reliably assigned to previously known (black) and novel (red) taxa, and branches that may belong to either known or novel taxa (grey).

Here we present a global-scale database assembled by the Global Spore Sampling Project (GSSP) that was initiated in 2018–2019^17^. The GSSP involves 47 sampling locations distributed across all continents except Antarctica, with each location collecting two 24-hr samples per week, in most cases over a period of one year or more (Fig. 1A,B). Sampling is conducted with a cyclone sampler, which orients itself in the direction of the wind. It collects particles >1 μm in size from the air directly into a sampling tube with a single reverse-flow cyclone. For DNA sequencing, we targeted part of the nuclear ribosomal internal transcribed spacer (ITS) region, which is the universal molecular barcode for fungi^18^.

To generate semi-quantitative estimates of DNA content (in units of ng of fungal DNA per m^3^ of air), we applied a spiking approach^17^ (Fig. 1C). To convert the sequence data into species data, we began by denoising the sequence yield into amplicon sequence variants (ASV^19^). We then applied probabilistic taxonomic placement using Protax-fungi^20,21^ to assign ASVs to taxa at ranks from phylum to species. Finally, we used a new constrained clustering approach (see *Methods*) guided by the taxonomic annotations from Protax-fungi to group ASVs into species-level operational taxonomic units (OTUs^22^). This clustering allowed us to assign OTUs to previously known and unknown taxa (Fig. 1D). Using a threshold of >90% probability of correct assignment, this resulted in 27,954 species-level OTUs, of which 1,392 could be reliably assigned to known species. The GSSP data are highly complementary to the Global Soil Mycobiome consortium (GSMc) data^10^, as among the 10 top ranking orders in the GSSP data, only 5 were found in the 10 top ranking orders of the GSMc data (Table 1).

**Table 1:** The most common orders found in the GSSP data and in the Global Soil Mycobiome consortium (GSMc) data^10^.

Dataset		GSSP		GSMc	
Phylum	Order	%	rank	%	rank
Ascomycota	Capnodiales	22.0	1	0.8	19
Ascomycota	Pleosporales	17.8	2	3.4	9
Basidiomycota	Polyporales	10.0	3	0.5	29
Basidiomycota	Agaricales	5.9	4	15.8	1
Basidiomycota	Tremellales	5.5	5	1.6	16
Ascomycota	Helotiales	4.2	6	6.3	3
Basidiomycota	Hymenochaetales	3.2	7	0.3	42
Ascomycota	Dothideales	2.4	8	0.2	50
Ascomycota	Eurotiales	2.0	9	4.3	7
Ascomycota	Chaetothyriales	1.8	10	2.8	10
Mortierellomycota	Mortierellales	0.06	75	6.3	2
Basidiomycota	Russulales	1.0	15	6.0	4
Basidiomycota	Thelephorales	0.08	63	5.8	5
Ascomycota	Hypocreales	1.0	14	5.0	6
Ascomycota	Pezizales	0.09	60	3.4	8

The table shows the relative abundance (%) of each order, computed as the mean across samples of the fraction of reads which were assigned to it, as well as the ranking of the order in terms of its abundance. Only orders that rank in the top ten in either of the two datasets are included.

## Methods

### Data acquisition

The Global Spore Sampling Project (GSSP) consists of a globally distributed network of 47 sampling sites collecting two 24-hr air samples per week over one to two years (Fig. 1). Each sampling site was equipped with a cyclone sampler (Burkard Cyclone Sampler for Field Operation, Burkard Manufacturing Co Ltd; http://burkard.co.uk/product/cyclone-sampler-for-field-operation). The sampling sites represent varying climatic zones and altitudes. Most sampling sites were located in natural environments, with a few in urban settings. Due to logistical reasons, we could not start the global sampling fully synchronously. In some locations, sampling had to stop earlier than expected due to external reasons (e.g., storms breaking the equipment or restrictions caused by COVID-19 lockdown). See Fig. 1 for realized sampling periods per site.

In October and November 2017, prior to the start of global sampling, a field test was performed in a grassy area at the University of Helsinki Viikki campus (60.2278 N, 25.01653E) to evaluate the quantity of fungal DNA collected over different time frames and in field blanks handled with and without the use of gloves on the part of the human handler. In total we collected seven 24-hour samples, three one-hour samples, and three 10-minute samples, in addition to four field blanks handled with gloves and five field blanks handled without gloves. For field blanks, Eppendorf vials were installed in the cyclone sampler in the field, but the sampler was not activated. The vials were then removed after one minute and sealed. Based on the results of these field tests (see *Technical Validation*), we decided to use a 24-hr sampling period, and to instruct the participating teams to handle the samples with gloves.

The functioning of the cyclone sampler and sample preparation procedure is described in detail in Ovaskainen *et al*.^17^. The cyclone samplers were placed at ground level to ensure free airflow through the sampler. The sampler collected particles >1 µm in size from the air directly into a sterile Eppendorf vial. The sampler’s average throughput of air was 16.5 L per minute for a total of 23,800 L (23.8 m^3^) during each 24-hour sampling period. After sampling, the vial was removed from the cyclone sampler, the lid was closed, and the vials were labelled with the site code and week number. We also recorded the time and duration of the sampling, along with notes on the presence of rainwater or larger objects (e.g., arthropods) in the sampling vial. To avoid contamination, gloves were used while handling the samples and the device. Participants were instructed to clean the cyclone part of the device monthly with water and soap and to rinse it with ethanol, or to sterilize it with dry-heat, chlorine, or UV when such equipment was available.

The samples were stored at −20 °C until shipped to the University of Helsinki, Finland. Shipping was done at room temperature. We do not expect much bias across samples due to this approach, as the shipping time was relatively short and most shipments were received with a similar delay. In Helsinki, the samples were separated from visible arthropods. To avoid losing fungal spores attached to arthropod bodies, the surface of any arthropod present in the sample was rinsed by adding sterile water into the sample tube and vortexing. After washing, the arthropods were removed with sterile tweezers. Samples containing any rainwater were dried in a vacuum drier (24 h). Prior to drying, each sample was covered with a porous Parafilm to avoid cross-contamination between samples. After drying, all samples were sent to the University of Guelph, Canada, for DNA extraction and sequencing.

### DNA extraction, sequencing, and quantifying DNA amount

A detailed description of DNA extraction, primers, and sequencing is given in Ovaskainen *et al*.^17^. In brief, the target genetic marker, i.e., the ITS2 region of the rRNA operon, was amplified using the polymerase chain reaction (PCR) for 20 cycles with fusion primers ITS_S2F^23^, ITS3, and ITS4^24^ tailed with Illumina adapters, and sequenced on Illumina MiSeq with 2 × 300 bp paired end reads. ITS_S2F was included as a second forward primer to specifically amplify plant DNA, in order to include pollen as well as fungal spores in the analysis. However, only a small fraction of reads resulted from the ITS_S2F-ITS4 amplicon, and so these were removed in the early stages of the analysis and not further considered. To quantify the amount of fungal DNA, we applied a spike-in approach^17^, using nine positive control plasmids prepared from synthetic sequences. These sequences were designed to be generally consistent with fungal ITS sequences, but different from all known natural sequences^25^. The positive synthetic control (0.01 ng/μl) containing nine plasmids was spiked into the PCR master mix at a ratio of 1:100 for the first 336 samples. For the remaining 2,432 samples, we used a 1:1000 ratio, since the 1:100 ratio produced an unnecessarily high proportion of the sequences representing the spikes. This could have compromised the sequencing depth of the targeted fungal sequences. We converted the ratio of the non-spike vs. spike-sequences into semi-quantitative estimates of DNA amount in units of ng of DNA per m^3^ of air as described previously^17^. The resulting estimates of DNA abundance correlated well with a qPCR-based estimate of DNA amount. Each MiSeq run included 84 study samples, one negative control sample introduced in the DNA extraction step, and two negative controls introduced in the PCR step. The only exceptions were two runs (CCDB-35004 and CCDB-35005) which included three extraction negative controls and no PCR negative controls. The same master mix as used for the study samples, including synthetic positive controls, was also used for the negative controls.

For the field test samples, DNA was extracted following the same protocol, except that 300 µL of ILB extraction buffer was used instead of 270 µL, and the final DNA extract was eluted into 35 µL of Tris buffer instead of 45 µL. Two extraction blanks were also included. A fungal DNA standard was extracted from Fleischmann’s Baker’s commercial yeast. Then, approximately one-half package of the commercial yeast was added to 50 mL warm water and proofed with sugar until the formation of active foam. Yeast DNA was extracted using an abbreviated version of the protocol described above, which omitted the initial ILB extraction buffer and homogenization in the TissueLyzer. Instead, six aliquots of 300 µL of yeast suspension were directly transferred to 900 µL each of 5 M GuSCN binding buffer, incubated at 56 °C for 1 hour in an orbital shaker, and then at 65 °C for 1 hour. The six eluates were pooled and quantified using a Qubit fluorometer with the DS DNA high sensitivity kit. The extract, which had a DNA concentration of 2.77 ng/µL, was then diluted to form standards of 1 ng/µL, 0.1 ng/µL, 0.01 ng/µL, 0.001 ng/µL, and 0.0001 ng/µL. The test samples were quantified by real-time PCR (RT-PCR) on a LightCycler96 (Roche) as described in Ovaskainen *et al*.^17^, with two replicates of each of the standards for calibration.

### Bioinformatic processing

Demultiplexed paired-end reads were first trimmed using Cutadapt version 4.2^26^. Because of low-quality base-calls at the 5′ end of R2 reads, we removed the first 16 bases from all R2 reads. We then trimmed the 3′ end of both reads with a quality threshold of 2 (i.e., remove only N’s), and the 5′ end of R2 with a quality threshold of 10. Reads were then trimmed to the ITS3-ITS4 amplicon, with a minimum 10 bp overlap and error tolerance of 0.2. Primers at the 3′ ends of both reads were optional but read pairs where the 5′ primer was not detected (including reads originating from the ITS_S2F-ITS4 amplicon) were removed. Pairs were discarded after trimming if either read was less than 100 bases or contained ambiguous bases. Reads were then further processed using DADA2 version 1.18.0^27^. First, all pairs where either read matched to the PhiX genome were removed, along with reads where R1 contained more than 3 expected errors or R2 contained more than 5 expected errors. Reads were denoised using separate error profiles fit for each MiSeq run with default parameters, and denoised read pairs were merged to form ASVs with a minimum overlap of 10 bp and a maximum mismatch of 1 bp. An initial de novo chimera check was performed on the merged ASV table using the DADA2 “consensus” method^27^. A second reference-based chimera check was then performed using the “uchime_ref” option in VSEARCH version 2.22.1^28^ with reference Sanger sequences from the UNITE v9database^29^, as used by the PlutoF Species Hypothesis matching pipeline^30^. The synthetic spike sequences were also included as references. Non-chimeric ASVs that were identical except for end gaps were combined, with the most abundant ASV sequence taken as representative. ASVs with a sequence similarity greater than 0.9 to SynMock spike sequences were identified using the “-usearch_global” command in VSEARCH 2.22.1^28^ and labelled as spike sequences. Non-spike sequences were aligned using Infernal 1.1.4^31^ to the covariance model for the combined 5.8 S and 28 S rRNA genes from the FunGene pipeline^32^ which was truncated to include only the region between the ITS3 and ITS4 primer sites. Sequences that did not match the full length of the model, or which scored less than 50, were discarded. This resulted in a 65,912 ASVs × 2,768 samples matrix, with entries representing read abundance.

A taxonomic affiliation was assigned to each non-spike ASV sequence using Protax-fungi^21^. This procedure gives assignments at each taxonomic rank from phylum to species, along with a calibrated probability that the assignment at each rank is correct. We used the 90% probability threshold for taxonomic assignments. Additionally, because Protax-fungi does not include non-fungi in its reference database, we matched ASVs to the same UNITE Sanger sequences mentioned above using the “usearch_global” command of VSEARCH 2.22.1^28^, with a sequence similarity threshold of 0.8. Sequences whose best match was annotated as belonging to a kingdom other than *Fungi*, or which had no match at the given threshold, were annotated as potential non-fungi but retained for the next clustering step.

Due to frequent intraspecific sequence variants for the ITS region, ITS-based ASVs are not suitable proxies for fungal species^33^. Consequently, we developed a taxonomically-guided clustering approach using the taxonomic annotations from Protax-fungi to group ASVs into approximately species-level OTUs. Our approach also groups sequences, including those without existing taxonomic annotations, into clusters approximating each taxonomic rank. First, we calculated optimal single-linkage clustering thresholds for each combination of a known taxon at a rank higher than species (henceforth, the “supertaxon”) and a taxonomic rank lower than that taxon (“subrank”) using multi-class F-measure optimization as described for the tool Dnabarcoder^34^. However, instead of using BLAST to calculate pairwise distances, as in Dnabarcoder, we based our clusters on a sparse pairwise sequence distance matrix generated by the -calc_distmx command in USEARCH 11.0.667^35^, with an initial kmer dissimilarity threshold of 0.4, maximum global alignment dissimilarity of 0.6, and a gap penalty of 1. For each supertaxon-subrank combination where there were at least five subtaxa represented by a total of at least ten reference sequences, we chose the clustering threshold that generated clusters most closely corresponding to the reference identifications. This match was assessed by the multi-class F-measure. Thus, we generated optimal thresholds for clustering all fungi into ranks from phylum to species; for clustering each phylum into ranks from class to species, and so on.

The ASVs were then clustered in three stages for each taxonomic rank from phylum to species, with the species-level clusters forming the final OTUs. In the first step, cluster cores were formed by the ASVs which had been assigned to taxa at that rank by Protax-fungi. These cluster cores were used as a reference for a closed-reference clustering stage, in which unassigned ASVs were matched to the closest cluster core using the optimized sequence similarity threshold for that rank and the nearest enclosing supertaxon. To this aim, we applied the “-usearch_global command” in VSEARCH version 2.22.1^28^. We used the same alignment penalties for closed-reference clustering as for the threshold optimization clustering above to ensure that distance calculations were comparable. Iterations were performed until no new matches were found, generating approximately single-linkage clusters without merging cluster cores. Finally, in the third step, remaining unclustered ASVs at each rank were clustered using de novo single-linkage clustering using distances calculated by USEARCH as above, and again using the optimized sequence similarity threshold for the rank and nearest supertaxon. These de novo clusters, which we refer to as “pseudotaxa”, were assigned placeholder taxonomic names of the form “pseudo{rank}_{number}” (e.g., “pseudogenus_0216” for a cluster at genus rank). At each taxonomic rank after phylum, the three clustering stages were performed within the clusters generated at higher taxonomic ranks. Thus, two ASVs that were assigned to, for instance, different phyla by Protax-fungi, could not be clustered together into the same pseudoclass, even when their sequence similarity was greater than the class-level threshold determined for one or both phyla.

Because the current version of Protax-fungi is trained only to identify fungi and not all eukaryotes, the non-fungal sequences were generally unidentified at the phylum level and were grouped into a large number of pseudophyla. We used the kingdom-level results from matching to the UNITE Sanger references (see above) to classify ASVs as “known fungi”, “known non-fungi”, or “unknown kingdom”, and removed pseudotaxa containing more known non-fungal ASVs than known fungal ASVs. At the phylum level, pseudotaxa containing only ASVs of unknown kingdoms were also removed.

The final result of this process was a 27,954 species-level OTUs × 2,768 samples read abundance matrix, along with taxonomic annotations at each rank from phylum to species, including pseudotaxon placeholders. The bioinformatics pipeline was implemented using the Targets package version 1.3^36^ in R version 4.2.2.

## Data Records

The database has been deposited to Zenodo^37^ and the sequence data are available at ENA European Nucleotide Archive^38^. The database is organized in five datasets in a csv format (columns separated by commas): (1) metadata providing the location, date, and time for each sample, along with sequencing depth and other essential information (Table 2); (2) species-level OTU tables per sample describing the number of sequences assigned to each species (Table 3); (3) taxonomic classification of each species-level OTU (Table 4); (4) closest matching sequences and their taxonomy for ASVs in putatively fungal pseudophyla, which are included in (2) and (3) (Table 5); and (5) closest matching sequences and their taxonomy for ASVs in putatively non-fungal pseudophyla, which are not included in the other datasets (Table 6). The first four datasets can be linked to each other using the unique sample codes and the unique identifiers for species-level OTUs.

**Table 2:** The fields of the metadata table (metadata.csv).

Field name	Description
sample.id	Unique identifier of the sample
seqrun	The run in which the sample was sequenced
site	The site of sampling
date	The year, month, and day of sampling
yday	The Julian day of sampling, ranging from 1 to 365
duration	The duration during which the sample was acquired, in hr
water	With levels “yes” if the sample contained water and “no.or.NA” if there was no water or the information was missing
insect	With levels “yes” if the sample contained insect(s) and “no.or.NA” if there were no insect(s) or the information was missing
unst.tweezers	With levels “yes” if the sample was processed with tweezers sterilized accidentally just by water and “no” if the tweezers were adequately sterilized
spike_dilution	The dilution level of the spike, either 0.01 or 0.001
numnonspikes	The number of sequences assigned to non-spikes
numspikes	The number of sequences assigned to spikes
dna_amount	The inferred total amount of fungal DNA in the sample (log_10_ transformed)
lat	Latitude of the site (decimal degrees)
lon	Longitude of the site (decimal degrees)
temp.mean	Mean annual temperature of the site (°C)

The rows of the metadata correspond to the samples.

**Table 3:** The fields of the samples x species-level OTU tables (otu.table.csv).

Field name	Description
sample.id	Unique identifier of the sample
Remaining fields	Unique OTU identifiers

The rows of the OTU tables correspond to the samples.

**Table 4:** The fields of the taxonomy tables (taxonomy.csv).

Field name	Description
OTU	Unique OTU identifier
nsample	The number of samples in which the taxon was found
nread	The total number of reads assigned to the taxon
kingdom	Inferred kingdom (always *Fungi*)
phylum	Inferred phylum
class	Inferred class
order	Inferred order
family	Inferred family
genus	Inferred genus
species	Inferred species
sequence	The sequence of the taxon

Levels of taxonomy that could not be reliably assigned to known taxa are indicated by names that include “pseudo”, numbered to allow identifying species that belong to the same unknown genus/family/order/class/phylum. The rows of the taxonomy tables correspond to the species-level OTUs.

**Table 5:** The fields of the fungal pseudophylum table (pseudophyla_fungi.csv).

Field name	Description
ASV	Unique ASV identifier
OTU	Unique identifier for the OTU which the ASV belongs to
pseudophylum	Unique identifier for the phylum-level cluster the ASV belongs to
pseudospecies	Unique identifier for the species-level cluster the ASV belongs to
sh_id	Unique identifier for the Unite species hypothesis (SH) of the best match to the ASV
dist	Sequence dissimilarity between the ASV and the best match. 0.0 = all bases identical, 1.0 = all bases different.
kingdom	Kingdom of the best matching sequence, as given in Unite
phylum	Phylum of the best matching sequence, as given in Unite
class	Class of the best matching sequence, as given in Unite
order	Order of the best matching sequence, as given in Unite
family	Family of the best matching sequence, as given in Unite
genus	Genus of the best matching sequence, as given in Unite
species	Species of the best matching sequence, as given in Unite

All amplicon sequence variants (ASVs) that could not be assigned to a fungal phylum, but which belong to a pseudophylum classified as *Fungi*, are included. These sequences are also represented as OTUs in the main OTU table and taxonomy. For each ASV, the closest matching species hypothesis (SH) in Unite is given, along with the classification of that sequence in Unite.

**Table 6:** The fields of the nonfungal pseudophylum table (pseudophyla_nonfungi.csv).

Field name	Description
ASV	Unique ASV identifier
pseudophylum	Unique identifier for the phylum-level cluster the ASV belongs to
pseudospecies	Unique identifier for the species-level cluster the ASV belongs to
sh_id	Unique identifier for the Unite species hypothesis (SH) of the best match to the ASV
dist	Sequence dissimilarity between the ASV and the best match. 0.0 = all bases identical, 1.0 = all bases different.
kingdom	Kingdom of the best matching sequence, as given in Unite
phylum	Phylum of the best matching sequence, as given in Unite
class	Class of the best matching sequence, as given in Unite
order	Order of the best matching sequence, as given in Unite
family	Family of the best matching sequence, as given in Unite
genus	Genus of the best matching sequence, as given in Unite
species	Species of the best matching sequence, as given in Unite

All amplicon sequence variants (ASVs) that could not be assigned to a fungal phylum, but which belong to a pseudophylum classified as non-*Fungi*, are included. These sequences are excluded from the main OTU table and taxonomy and so do not have OTU identifiers. For each ASV, the closest matching species hypothesis (SH) in Unite is given, along with the classification of that sequence in Unite.

## Technical Validation

### Field tests and negative controls

**Fig. 2:**
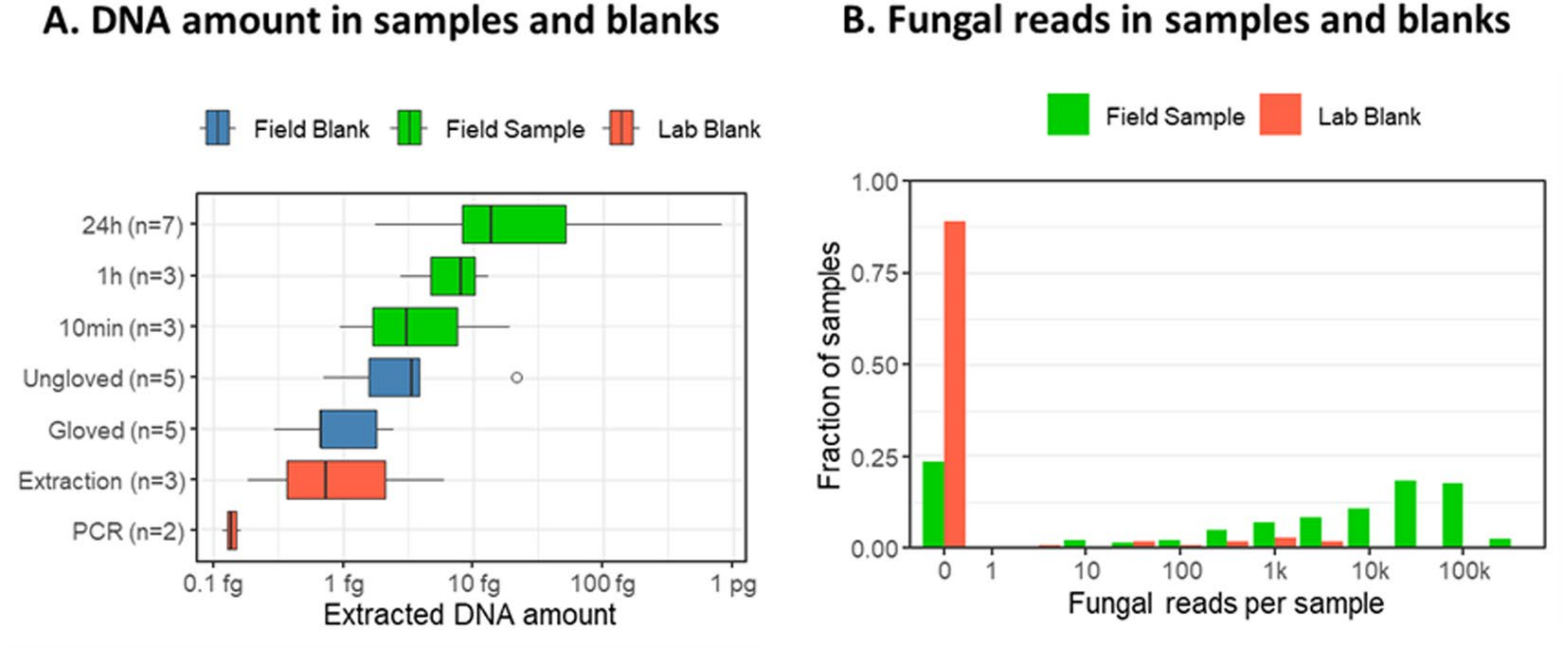
Results from field tests and negative controls. Panel A shows DNA concentration in the field test samples based on either 24-hr sampling, 1-hr sampling, or 10-min sampling, as PCR blanks, extraction blanks, and field blanks handled with and without gloves. Panel B shows the distributions of the number of fungal reads per sample based on either field samples (green bars), field blanks (blue bars), or lab blanks (red bars). Note the logarithmic scale in the x-axis.

The median DNA amount measured by RT-PCR in the seven 24-hour test samples was 14 fg of DNA. The median DNA content measured in 1-hour samples was 8 fg, and the median for 10-minute samples, as well as for field blanks handled without gloves, were less than 3 fg. The median DNA quantity measured in the field blanks handled with gloves and the extraction blanks were approximately 0.7 fg, and the DNA quantity in the PCR blank was approximately 0.1 fg (Fig. 2A). As these values were standardized using genomic DNA extracted from yeast, they cannot be directly translated to other fungi due to varying genome size and ITS copy number. Nonetheless, we note that 24-hour field samples had almost 5 times more ITS copies than blank samples handled without gloves, and twenty times more than blank samples handled with gloves. In the actual study, all samples were handled with gloves.

Of the 99 negative controls, 89% of samples (i.e., 88 samples) did not yield any reads of fungal origin at the end of the bioinformatic analysis. For all sequencing runs, at least one negative control sample contained 0 fungal reads, indicating that the reagents were uncontaminated. The 9 negative control samples that did produce fungal reads yielded fewer fungal reads than the study samples (Fig. 2B), and, in most cases, these reads belonged to only one or two OTUs. OTUs found in negative control samples were all relatively common in the study. They were no more common in the sequencing runs which contained the negative controls than in other sequencing runs. This suggests that the most likely source of these reads was infrequent cross-contamination from study samples to negative controls. Among the negative controls, sample CCDB-35071NEGPCR2 yielded the highest read count: 2,668 fungal reads. All 18 OTUs detected in this sample were also found in sample COR_41A with abundances 7–60 times as high as in the negative control. Samples CCDB-35071NEGPCR2 and COR_41A were processed in the same sequencing run, indicating that the sample COR_41A was likely the source of cross-contamination.

### Sufficiency of sequencing depth

**Fig. 3:**
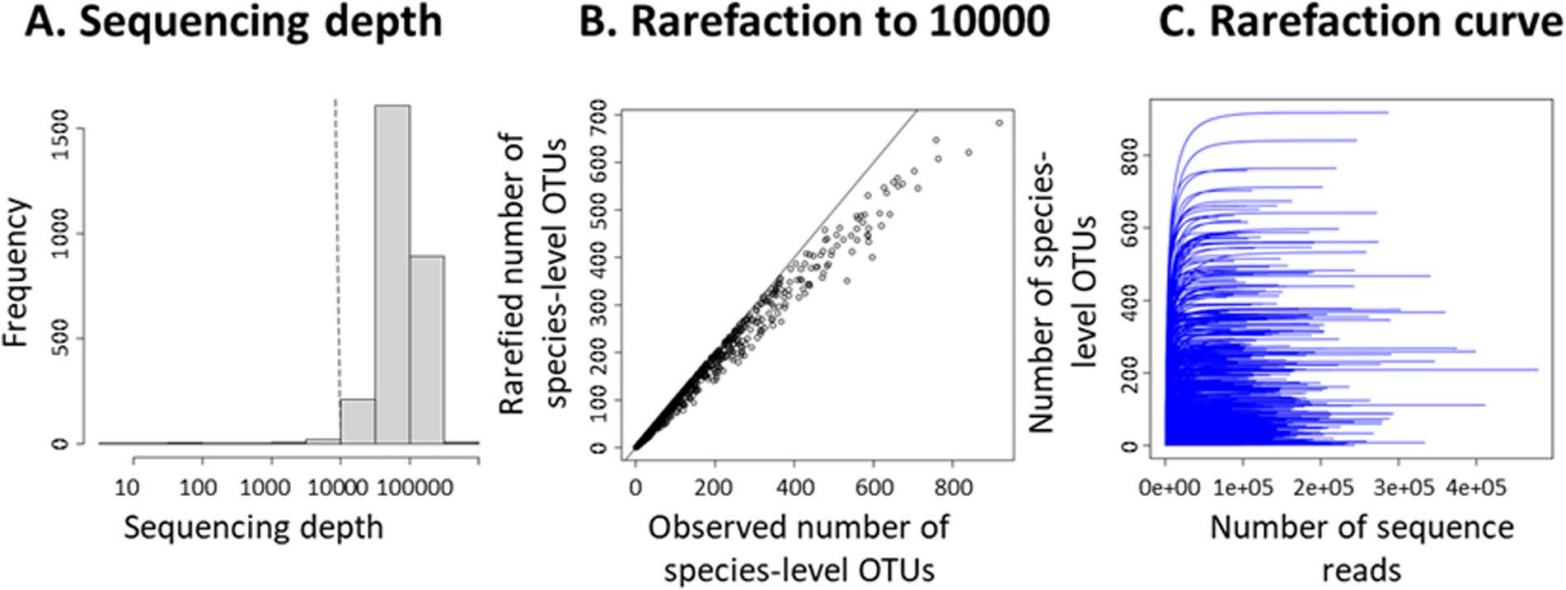
Results illustrating the sufficiency of sequencing depth, i.e., the total number of sequencing reads (including fungal and spike reads) obtained for each sample. Panel A shows the distribution of sequencing depth among the samples, with the dashed vertical line corresponding to the value of 10,000 sequence reads, which we recommend using as a threshold for including a sample for analyses. Panel B shows the decrease in the number of species-level OTUs if rarefying all samples to 10,000 sequence reads. Panel C shows rarefaction curves for all samples that included at least 10,000 sequence reads.

The mean sequencing depth among the samples was 86,845, and the median sequencing depth was 79,396. We recommend conducting analyses with samples yielding at least 10,000 sequencing reads, which corresponds to discarding 50 samples and thus 1.8% of the samples (Fig. 3A). If rarefying all samples to 10,000 sequence reads, a minor loss of species-level OTU richness is observed for the most diverse samples (Fig. 3B). Nonetheless, even the most diverse samples were likely sequenced to an adequate depth, as illustrated by the well-saturating rarefaction curves (Fig. 3C).

### Validation of automated taxonomic classifications by manual expert evaluation

Molecular taxonomic identification of fungi from environmental samples is challenging for several reasons^21^. First, the diversity of fungi is enormous, and most species are still unknown to science. Second, reference sequences are available only for a subset of the scientifically described species. Third, the systematics of fungi remains partially or even largely unresolved and undergoes continuous revisions. Fourth, the reference sequences in standard databases contain errors, and a substantial proportion of the reference sequences are mislabelled. Fifth, unlike the COI region used for molecular identification of animals, the ITS region does not allow for alignment at deep phylogenetic scales (much above the genus level), making sequence comparison more challenging. PROTAX-fungi explicitly accounts for all these sources of uncertainty while performing probabilistic taxonomic classification, and its validity has been tested by cross-validation experiments^21^.

Given the taxonomic breadth of the data and the unexplored nature of airborne fungal diversity, we evaluated the validity of the PROTAX classifications by comparing them to taxonomic classifications carried out by independent experts. To do so, we first clustered the sequences with 97% similarity threshold and selected the most common sequence in each cluster as its representative. We then selected a total of 500 clusters (and their corresponding representatives) as follows: (i) 200 sequences that PROTAX could not reliably (with at least 90% probability) classify to any known phylum, in which case they are unlikely to belong to the fungal kingdom; (ii) 50 sequences that PROTAX reliably classified to a known phylum but an unknown class; (iii) 50 sequences that were reliably classified to a known class but an unknown order; (iv) 50 sequences reliably classified to a known order but an unknown family; (v) 50 sequences reliably classified to a known family but an unknown genus; (vi) 50 sequences reliably classified to a known genus but an unknown species; and (vii) 50 sequences reliably classified to a known species. Within each category, we selected clusters that achieved the highest prevalence (i.e., that occurred in the highest proportions of the samples) in the GSSP data. Two authors with fungal taxonomic expertise (Otto Miettinen and Anton Savchenko) then manually performed the taxonomic classification of these 500 sequences, up to the taxonomic resolution that they considered possible to reliably achieve. The expert assessment was based on the first 100 BLAST hits between the query sequence and reference sequences in publicly available gene databases, thus incorporating a larger body of information than just a few top hits. In their assessment, the experts accounted for the quality issues in the reference sequences, such as divergent tail regions in poorly trimmed Sanger sequences, or chimeric sequences. Furthermore, naming of the sequences varies wildly, and experts used their judgement on which sequences to trust as the reference, and to what degree. There might be equally good hits under several names, in which case the experts judged which one was most likely correct. The best hit might refer to a name that is a collective, not allowing species-level identification with certainty. An important criterion in judging the reliability of reference sequences was related to the perceived trustworthiness of the sequence authors based on their taxonomic expertise (i.e., their standing in the field). As there is no published, up-to-date taxonomy for all fungal taxa, in many cases the experts had access to more up-to-date information (e.g., unpublished sources) about the classification, and then used this information when deciding on the correct naming at all taxonomic ranks.

The taxonomic experts knew the criteria used to select the sequences, whereas the order in which the sequences were provided was randomized, so that the experts did not have *a priori* information about the PROTAX classifications. We compared the classifications achieved by PROTAX versus the experts by computing the numbers of consistent and inconsistent classifications for each taxonomic level. The consistent and inconsistent classifications were counted separately for each of the following four confidence levels of PROTAX identifications: reliable identifications (i.e., those with at least 90% probability of correct classification), plausible identifications (those with at least 50% but less than 90% probability of correct classification), best hits (the classification with highest probability, where the highest probability is at least 1% but less than 50%), and no hits (those for which PROTAX did not yield any classification with at least 1% probability).

**Fig. 4:**
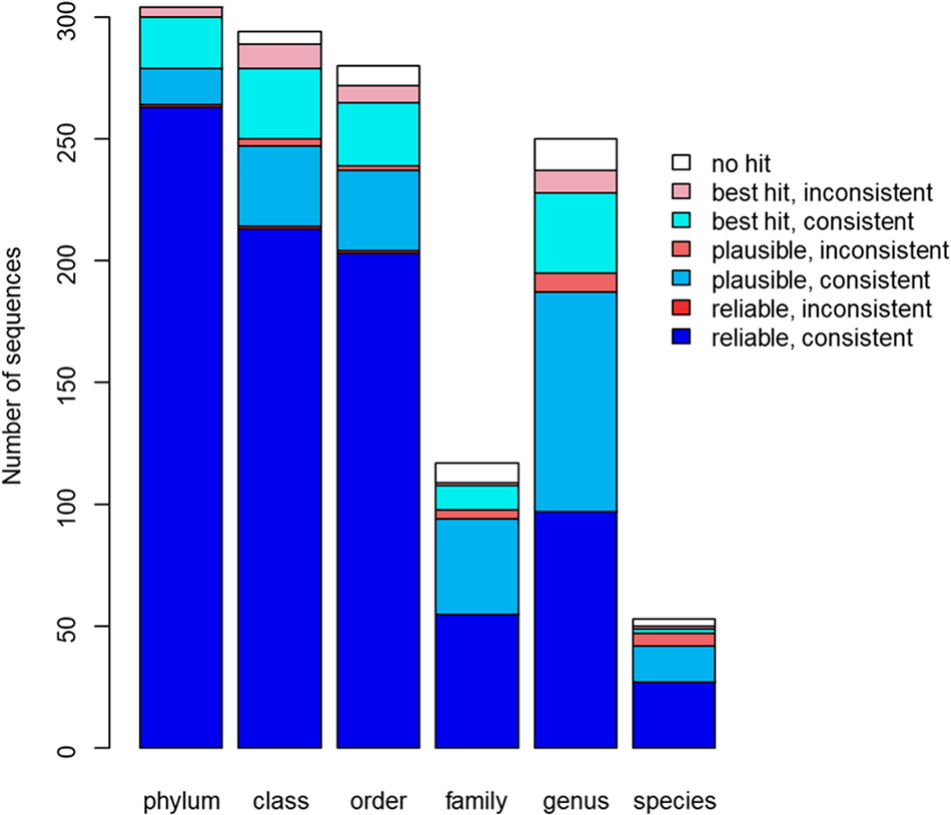
Comparison between PROTAX and expert classifications. The bars correspond to sequences that experts classified to at least the level of phylum, class, order, family, genus, or species. Blue colours correspond to cases where PROTAX yielded a classification consistent with the expert classification, and red colours to cases where PROTAX yielded an inconsistent classification. The brightness of the colour indicates the level of reliability in the PROTAX identification (reliable or plausible, see legend). We note that the number of families is smaller than that of the genera, because we have excluded cases where the experts did not provide a classification at the family level. Such apparent inconsistencies will appear for the many fungal orders where there are no well-established family-level classifications. In these cases, the genera are placed directly under the orders.

PROTAX-fungi classifications and expert classifications were highly consistent (Fig. 4). Most importantly, out of those 861 cases where PROTAX yielded a reliable classification at a given rank, the classification differed from that of the experts in only three cases (0.35% of the cases). Out of the 247 cases for which PROTAX yielded a plausible classification, the classification differed from that of the experts in 9% of the cases. Out of the 154 cases where PROTAX yielded merely a best hit, the classification differed from that of the experts in 21% of the cases. Out of those 189 cases that the experts classified as belonging to groups other than fungi (48 cases of Viridiplantae and 14 cases of Metazoa) or found impossible to reliably classify as fungi, PROTAX never produced a reliable phylum-level classification.

Figure 4 shows only cases where the experts classified the sequences to at least the same taxonomic level as did PROTAX. However, there were also 29 cases for which the experts considered it possible to reliably classify the sequence up to the genus level, but PROTAX provided a reliable classification to the species level. Out of these 29 cases, the experts gave an uncertain species-level classification for 15 cases. In each of these cases, the classification offered by the experts was consistent with the classification provided by PROTAX. In addition, there was one case in which the experts provided only a class-level classification and one case where the experts gave an order-level classification, but PROTAX considered it possible to reliably provide also more resolved classifications.

Based on these results, we conclude that the taxonomic classifications provided by PROTAX are highly consistent with those carried out manually by experts, but that PROTAX is generally more conservative regarding the reliability of the classifications. The difference in the uncertainty assessment is at least partially due to the fact that PROTAX explicitly accounts for the possibility that the sequence represents an unknown taxon – and such taxa are likely to be common in the global aerial data. As the manual classifications involved only a negligible fraction of all the sequences, the classifications published in the database were conducted by PROTAX.

### Validation of automated taxonomic classifications by comparison with the Global Biodiversity Information Facility (GBIF) database

To further validate the reliability of the automated taxonomic classifications, we compared the spatial distributions observed in this study to species occurrence records present in the Global Biodiversity Information Facility (GBIF) database. The motivation behind this comparison was to assess how likely the taxonomic classifications based on DNA barcoding match with classifications conducted by earlier research – as based mostly on morphological characters. To evaluate this consistency, we compared the spatial distributions of species recorded in this study to those recorded in the GBIF database. Cases where a difference in the distributions recorded suggested an error in the taxonomic classification were then examined in greater detail. To download occurrence records from GBIF, we used the function *occ_download* of the R-package *rgbif* v3.7.7 with R-version 4.3.1 for the 1,319 species that were reliably identified in our data, and for which occurrence data was available in GBIF (GBIF.org. 27 August 2023, GBIF Occurrence Download DOI 10.15468/dl.t8yn8x, with 6,189,602 occurrences).

Quantifying the consistency between our GSSP data and GBIF data is not straightforward, because the GBIF data is presence-only in nature without a well-controlled observation effort. To avoid biasing the results due to uncontrolled variation in sampling effort among species and across space in the GBIF data, we applied a null-model approach. Here, we constructed a null distribution that described the consistency between the spatial distribution of each focal species in the GBIF database and of all non-focal species in the GSSP data. For GSSP data, we used the prevalence of a species *p*_*i*_ (i.e., fraction of samples in which the species was present) as the measure of species abundance for each site *i*. For the GBIF data, we computed a GBIF-index *g*_*i*_ describing how frequently the species was observed in the proximity of the site *i* for each of our sampling sites. To do so, we defined *g*_*i*_ as the weighted sum over all GBIF occurrences where we weighted each occurrence by $\exp \left(\frac{-d}{1000}\right)$, where *d* is the distance (in kilometers) between the focal site *i* and the location of the GBIF occurrence. As a measure of consistency between the spatial distributions in the two datasets, we then computed the correlation between *p*_*i*_ and *g*_*i*_ over the sites. For each focal species, the observed value is the consistency between the focal species in the GBIF data and the focal species in our data, whereas the null distribution encapsulates the consistencies between the focal species in the GBIF data and all non-focal species in the GSSP data. As an empirical p-value, we computed the proportion of the null distribution instances where the value exceeded the observed one. This comparison was carried out for 1,251 out of the 1,319 species, since for 68 species the number of datapoints was too low, resulting in a NA value for the correlation.

**Fig. 5:**
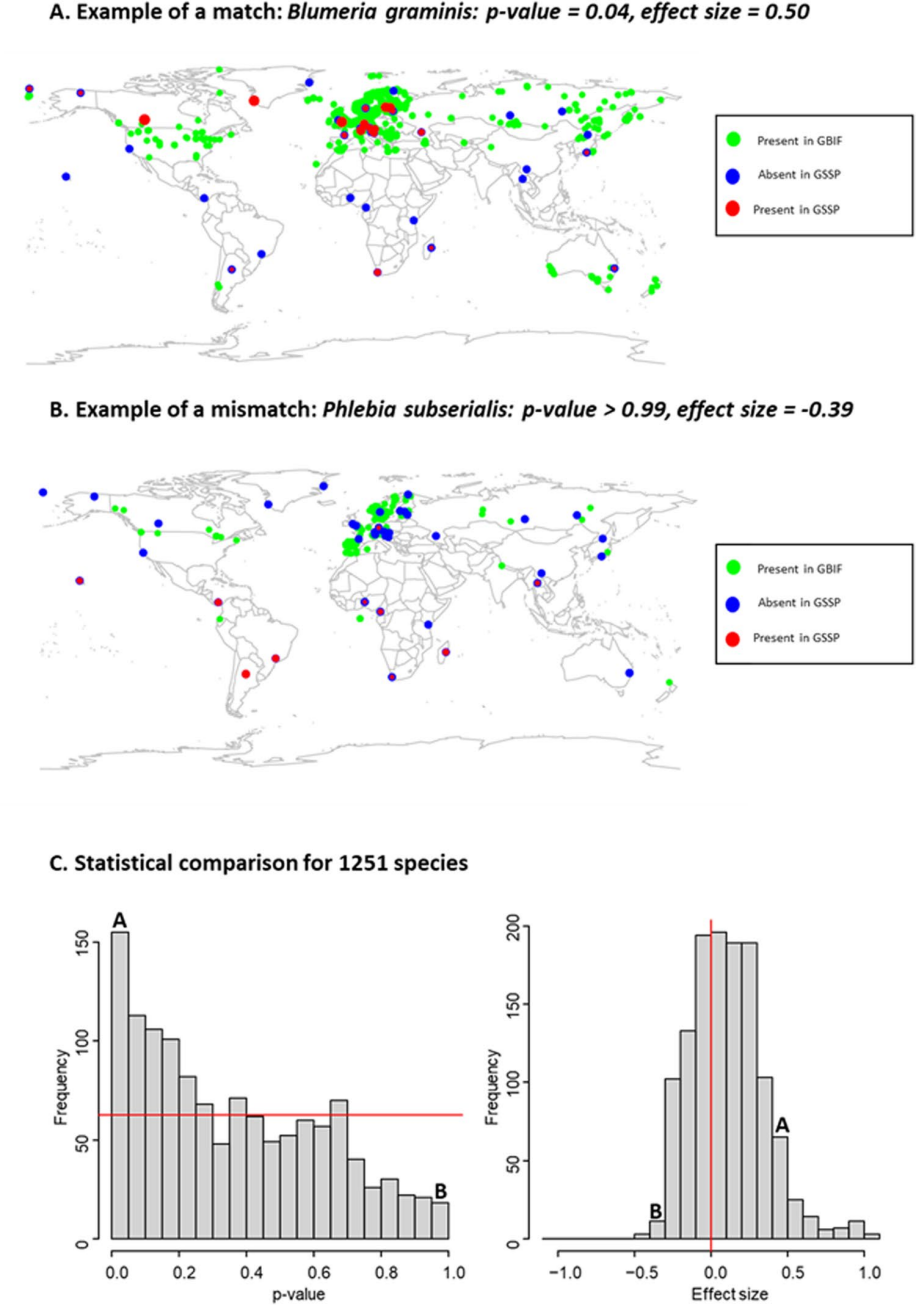
Comparison between GSSP and GBIF data. The upper panels show a visual comparison between GSSP data and GBIF data exemplified for a species with a match better than expected at random (panel **A**: *Blumeria graminis*, correlation = 0.50, p-value 0.04), and for a species with a match worse than expected at random (panel **B**: *Phlebia subserialis*, correlation = −0.39, p-value > 0.99). For GBIF data, all occurrence records are shown in green circles. For GSSP data, all sampling locations are indicated as a blue circle, including locations where the species was not observed. In locations where the species was observed, the size of the red circle shows the proportion of samples in which the species was observed. The lower panels (**C**) show a statistical comparison for all 1,251 species included in the analysis. The *p*-value shows the statistical significance of the comparison, with small *p*-values corresponding to cases where the GBIF data for the focal species was more consistent with the GSSP data for the focal species than with the GSSP data for a randomly selected non-focal species. The effect size shows the correlation between the GBIF data and GSSP data for each focal species. In both panels, the red line highlights the null expectation based on no consistency between the GBIF and GSSP dataset, indicating that for the majority of the species, the GBIF and GSSP datasets match much better in their spatial distributions than expected by random. The frequency bins into which the species exemplified in panels A and B fall are highlighted with letters A and B in panel C.

Overall, the species distributions revealed by our study were consistent with their known distributions in the GBIF database – in the sense that their distributions in the GSSP data coincide more with the distributions in GBIF than with random distributions (Fig. 5). This comparison also highlights the large number of species for which the match is no better than random (as revealed by *p*-values in the range from 0.05 to 0.95 in Fig. 5C). This lack of statistically significant matches was expected, as the GBIF data on most fungal species derive from opportunistic observations rather than from systematic surveys. The comparison further highlighted 14 species (*Cystobasidium minuta, Sphaerobolus ingoldii, Gaeumannomyces graminis, Phialemonium dimorphosporum, Xenasmatella ardosiaca, Zygoascus hellenicus, Meyerozyma guilliermondii, Candida intermedia, Trametes polyzona, Lodderomyces elongisporus, Hansfordia pulvinata, Physisporinus vitreus, Scopuloides rimosa* and *Phlebia subserialis*) for which the match was worse than expected by random (p-value > 0.95). While the proportion of such mismatches are less than expected by chance (since a uniform distribution of p-values would lead to 63 such cases), this list identifies candidates for misclassification and were thus examined manually in more detail.

For two of the mismatches, the inconsistency was most likely explained by erroneous records in GBIF: OTUs classified here as *Phlebia subserialis* and *Sphaerobolus ingoldii*. The name *P. subserialis* is known to have been applied to multiple biological species of corticioid wood decay fungus that are morphologically similar but not very closely related^39,40^, likely creating erroneous records in GBIF (Fig. 5B). The wood-decaying fungus *Sphaerobolus ingoldii* was described in the 21^st^ century based on DNA evidence, and it is morphologically similar to *S. stellatus*^41^. We thus assume that the old GBIF observations of *S. stellatus* in South Africa and Australia might be *S. ingoldii* instead.

For three of the mismatches, we considered the name assigned in GSSP incorrect: OTUs classified here as *Phialemonium dimorphosporum, Physisporinus vitreus*, and *Scopuloides rimosa*. For these cases, there were either exactly matching reference sequences representing multiple species, or there was divergence among the PROTAX assignments of the ASVs that were included in the OTU. Thus, in these cases, the classification selected by our algorithm was somewhat ambiguous, even when at least one of the ASVs belonging to the OTU cluster achieved at least 90% probability of correct classification.

For two of the mismatches (*Xenasmatella ardosiaca* and *Trametes polyzona*), our manual inspection revealed that we had accidentally imported an incorrect species from GBIF (or only partial data for the focal species), whereas the correct data from GBIF actually showed a good match with the GSSP records. Hence, only 12 (not 14) species in the end showed a mismatch between the two databases. However, to keep our technical validation transparent and to point out the range of errors that may take place in automated comparisons, we decided to report on these two apparent mismatches here. For the remaining seven mismatches (*Cystobasidium minuta, Gaeumannomyces graminis, Zygoascus hellenicus, Meyerozyma guilliermondii, Candida intermedia, Lodderomyces elongisporus*, and *Hansfordia pulvinatae*), our manual inspection suggested that there was indeed a mismatch between the GSSP and GBIF distributions, but it was difficult to judge whether the problem was in the GSSP classifications, in the GBIF records, or in both of these, highlighting another common issue in automated comparisons.

From the comparison between GSSP and GBIF, we conclude that both molecular and morphological classifications of fungi are challenging. Both databases are indeed likely to have some level of error, especially at the species level. Yet, even at the species level, a high proportion of the cases supported the validity of both the GSSP and GBIF data by showing that they match better than expected at random. Only for 1% of the cases (12 out of 1,251) did we find a mismatch that was significant at the p < 0.05 level; the comparison thus supports the technical validity of the GSSP data.

### Affinity of sequences which could not be assigned to fungal phyla

As described above, ASV sequences that could not be assigned to a fungal phylum either by Protax with probability >90% or by clustering with other ASVs which were so assigned by Protax were de novo clustered into “pseudophyla”. These pseudophyla are expected to contain real fungal sequences which lack close matches in the Protax reference database, as well as real non-fungal sequences and sequencing artifacts. Because we are unable to draw confident conclusions about the taxonomic affinity of these pseudophyla on the basis of the Protax results, we have included data tables providing, for each ASV in each pseudophylum, information on the closest matching species hypothesis (SH) in the Unite Sanger reference database^29^, the sequence dissimilarity of that closest match as calculated by VSEARCH, and the taxonomy given in Unite (the “best-hit taxonomy”). Although we do not consider the best-hit taxonomy to be reliable without extensive manual validation, we also summarize the best-hit taxonomy at the phylum level for likely fungal pseudophyla (Table 7) and at the kingdom level for likely non-fungal pseudophyla (Table 8). In almost all cases, multiple pseudophyla share the same best-hit taxonomy; however, the best-hit taxonomy within each pseudophylum is quite consistent, as indicated by low numbers of “minority” ASVs, especially within the fungi. This suggests that pseudophyla (and presumably other pseudotaxa, at least at higher taxonomic ranks) are most likely underclustered, in the sense that two sequences which are in the same pseudophylum can be confidently assumed to belong to the same phylum, while sequences in different pseudophyla cannot be so confidently assumed to belong to different phyla. Although many pseudophyla include multiple ASVs that cluster into multiple pseudospecies, we note that the 738 pseudophyla with no match of less than 20% sequence dissimilarity (Table 8) each contains exactly one pseudospecies, although in some cases these pseudospecies do consist of multiple ASVs. We suggest that the sequences included in these pseudophyla, which like the rest of the non-*Fungi* pseudophyla are not included in the main data tables, are particularly likely to be sequencing artifacts, although some highly divergent unknown taxa may also be included.

**Table 7:** Summary of closest Unite matches for pseudophyla included in kingdom *Fungi*.

Majority phylum	# pphy	# psp	ASVs	Mean distance
*Basidiomycota*	22	131	191 (135/0/2/53)	0.101/–/0.019
unspecified *Fungi*	60	101	178 (0/21/155/2)	–/0.021/0.044
*Chytridiomycota*	56	115	146 (131/0/9/0)	0.052/–/0.038
*Ascomycota*	35	71	102 (81/3/13/0)	0.051/0.079/0.059
*Rozellomycota*	24	57	62 (50/0/4/1)	0.027/–/0.006
*Blastocladiomycota*	9	33	34 (32/0/0/0)	0.096/–/–
*Olpidiomycota*	4	13	32 (27/0/0/0)	0.023/–/–
*Aphelidiomycota*	6	13	20 (20/0/0/0)	0.062/–/–
*Mucoromycota*	5	5	9 (9/0/0/0)	0.007/–/–
*Monoblepharomycota*	3	3	3 (3/0/0/0)	0.051/–/–
*Zoopagomycota*	2	2	2 (2/0/0/0)	0.087/–/–

Each row summarizes pseudophyla according to the most common best-hit phylum (“Majority phylum”) among their constituent ASVs. ASV counts are given as “total (majority/minority/unspecified/no match)”, where “total” is the number of ASVs included in all such pseudophyla, “majority” is the number of ASVS whose best-hit phylum is the majority phylum, “minority” is the number of ASVs whose best-hit phylum is a different named phylum, “unspecified” is the number of ASVs whose best-hit sequence is not identified at the phylum level (i.e. “Fungi_phy_unspecified”), and “no match” is the number of ASVs which had no match at a 20% global dissimilarity threshold. Mean distance is similarly given as “majority/minority/unspecified”. No mean distance can be calculated for ASVS in the “no match” category. Abbreviations: pphy = pseudophyla, psp = pseudospecies.

**Table 8:** Summary of closest Unite matches for pseudophyla not included in kingdom *Fungi*.

Majority kingdom	# pphy	# psp	ASVs	Mean distance
*Viridiplantae*	105	934	3572 (3016/174/331/51)	0.023/0.063/0.027
no match	738	738	1577 (0/0/0/1577)	–/–/–
*Alveolata*	29	105	1553 (1050/4/396/103)	0.051/0.017/0.060
unspecified Eukaryote	222	272	958 (0/124/629/205)	–/0.072/0.085
*Rhizaria*	48	83	360 (139/56/163/2)	0.036/0.075/0.052
*Metazoa*	49	75	312 (246/0/50/16)	0.089/–/0.098
*Stramenopila*	24	35	142 (99/1/34/8)	0.066/0.006/0.065
*Amoebozoa*	3	5	21 (4/0/17/0)	0.022/–/0.047
*Cryptista*	4	7	15 (4/4/4/3)	0.023/0.029/0.041
*Heterolobosa*	1	2	7 (6/1/0/0)	0.024/0.003/–
*Planomonada*	1	1	2 (2/0/0/0)	0.090/–/–
*Apusozoa*	1	1	2 (2/0/0/0)	0.010/–/–
*Glaucocystoplantae*	2	2	2 (2/0/0/0)	0.007/–/–

Each row summarizes pseudophyla according to the most common best-hit kingdom (“Majority kingdom”) among their constituent ASVs. ASV counts are given as “total (majority/minority/unspecified/no match)”, where “total” is the number of ASVs included in all such pseudophyla, “majority” is the number of ASVS whose best-hit kingdom is the majority kingdom, “minority” is the number of ASVs whose best-hit kingdom is a different named kingdom, “unspecified” is the number of ASVs whose best-hit sequence is not identified at the phylum level (i.e. “Eukaryota_kgd_unspecified”), and “no match” is the number of ASVs which had no match at a 20% global dissimilarity threshold. Mean distance is similarly given as “majority/minority/unspecified”. No mean distance can be calculated for ASVS in the “no match” category. Abbreviations: pphy = pseudophyla, psp = pseudospecies.

### Main sources of variation in the data

To evaluate the types of ecological signals present in the data, we quantified the main sources of variation. We fitted a generalized linear model to a data set including each 485 species-level OTU that occurred at least 50 times in the data. We truncated the data to presence-absence and applied probit regression with the R-package Hmsc^42^. As fixed effects, we included log(sequencing depth), the mean temperature of the site and its square, and the interaction between latitude and seasonality. We modelled “seasonality” with the periodic functions $\sin \left(2\pi \frac{d}{365}\right)$ and $\cos \left(2\pi \frac{d}{365}\right)$, where *d* is the Julian day of the year. As latitude is positive for the Northern and negative for the Southern Hemisphere, we note that the interaction between seasonality and latitude appropriately assumes opposite patterns of seasonality in the two hemispheres. To capture spatial variation not captured by the annual mean air temperature of the site, we included the site as a random effect. We assumed the default prior distributions of Hmsc^43^ and fitted the models using the Markov Chain Monte Carlo (MCMC) procedure^42^. We included four MCMC chains with 37,500 iterations in each, out of which we discarded 12,500 as transient and thinned the remaining iterations by 100, obtaining 250 posterior samples per chain and hence 1,000 posterior samples in total. We followed Tikhonov *et al*.^42^ to evaluate the models’ explanatory power with Tjur’s *R*^2^ and AUC and partitioned the explained variation to its components explained by temperature, seasonality, sequencing depth, and the random effect of the site.

The models achieved a satisfactory model fit, with mean (over the species) AUC = 0.91 and mean Tjur’s *R*^2^ = 0.18. The annual mean air temperature of the site explained the largest portion of the variation (53%, averaged over the species), followed by the random effect of the site (29%), seasonality (12%), and sequencing depth (5%). These results suggest that the data contain a strong ecological signal, as species distributions are strongly structured by space – in particular by the annual mean air temperature of the site.

## Data Availability

The data, the bioinformatics pipeline, and the R-pipeline that performs the technical validation are available in Zenodo^37^.
